# Dietary L-Malic Acid Enhances Growth Performance and Oxidative Myofiber Composition in Nursery Piglets

**DOI:** 10.3390/molecules31193381

**Published:** 2026-09-23

**Authors:** Qian Zhu, Hanyu Hu, Md. Abul Kalam Azad, Haoran Wang, Gurui Gao, Xiangfeng Kong

**Affiliations:** 1 Hunan Provincial Key Laboratory of Animal Nutritional Physiology and Metabolic Process, National Engineering Laboratory for Pollution Control and Waste Utilization in Livestock and Poultry Production, Institute of Subtropical Agriculture, Chinese Academy of Sciences, Changsha 410125, China; walanzhuqian@163.com (Q.Z.); 13866299154@163.com (H.H.); azad.fpe@gmail.com (M.A.K.A.); wanghaoran241@mails.ucas.ac.cn (H.W.); gaogurui1895@163.com (G.G.); 2 Hunan Provincial Key Laboratory of the Traditional Chinese Medicine Agricultural Biogenomics, Changsha Medical University, Changsha 410219, China; 3 College of Animal Science and Technology, Guangxi University, Nanning 530004, China; 4 College of Advanced Agricultural Sciences, University of Chinese Academy of Sciences, Beijing 100049, China

**Keywords:** AMPK/PGC-1α signaling pathway, antioxidant capacity, energy metabolism, L-malic acid, mitochondrial biogenesis, muscle fiber types, functional nutrition, nursery piglets

## Abstract

L-malic acid (L-MA) is a naturally occurring organic acid abundant in fruits and traditional plant-based foods. L-MA, chemically identical to the natural form, has potential for optimizing energy metabolism and strengthening the antioxidant system in animal production. **Objective:** This research evaluated the impacts of dietary L-MA on nursery piglets’ growth performance, carcass traits, and muscle development. Forty castrated male crossbred piglets (BW: 8.06 ± 0.43 kg) were randomly assigned into five groups and provided a basal diet containing 0%, 0.25%, 0.5%, 1%, or 2% L-MA for a 56-day feeding trial. Dietary 0.5–2% L-MA enhanced final BW, ADG, and ADFI. All L-MA (0.25–2%) groups showed increased carcass weight, muscle length, and loin eye area, as well as higher pH value and color, and reduced drip loss and cooking loss of muscle tissues. An increased muscular abundance of oxidative fibers was observed in response to L-MA, accompanied by upregulated *MyHCI* and *MyHCIIa* and downregulated glycolytic-fiber-related gene expressions. Additionally, L-MA enhanced muscular SOD, GSH-Px, and CAT activities and upregulated *SOD*, *GSH-Px*, *CAT*, and *NRF2* expressions, accompanied by a reduced MDA content. Furthermore, L-MA elevated plasma and muscular SDH and MDH activities and suppressed LDH activity. Moreover, L-MA augmented transcription of the myogenic markers (*Myf6* and *MyoD*) and mitochondrial-related genes (*COX4*, *Cytc*, *TFAM*, *NRF1*, *PGC-1α*, and *Sirt1*), increased mitochondrial abundance, as evidenced by enhanced TOM20 intensity and upregulated expressions of *COX4*, *Cytc*, *TFAM*, *NRF1*, *PGC-1α*, and *Sirt1*. These changes were accompanied by transcriptional alterations in the AMPK/SIRT1/PGC-1α/NRF1 signaling axis. Significant enhancement effects were observed at L-MA supplementation levels of 0.5% and 2%. In summary, dietary L-MA improves ADG, ADFI, and final BW of nursery piglets, as well as muscle characteristics relevant to subsequent pork quality by enhancing antioxidant capacity, stimulating oxidative metabolism, and promoting slow muscle fiber formation. The observed effects may be partially associated with the AMPK/PGC-1α signaling pathway at the transcriptional level, as well as an increase in mitochondrial abundance. While these findings suggest a potential enhancement of mitochondrial biogenesis, definitive proof would require functional assays. Collectively, these findings underpin the potential of L-MA as a functional nutrient, a natural-identical source for improving performance and muscle quality characteristics in pig production systems. In addition, this study also contributes to the development of high-quality pork.

## 1. Introduction

High-quality animal protein, particularly pork, is not only a matter of palatability but also closely linked to consumer health. In recent years, high-quality pork has attracted growing consumer interest, leading to an imbalance between supply and demand. As a result, practitioners in the pig industry have been continuously developing and promoting technologies to improve pork quality. Muscle fibers are the fundamental structural units of muscle tissue, and their types and composition not only determine skeletal muscle development but also affect pork quality [1]. Muscle fiber development is mainly divided into prenatal and postnatal stages. Muscle fiber quantity is primarily set by the 90th day of prenatal development, whereas postnatal development primarily involves fiber-type transformation and hypertrophy [2]. It is noteworthy that the period from birth to the 70th day represents a critical phase for changes in muscle fiber types [3]. Therefore, muscle fiber type is an important regulatory target for improving pork quality and producing high-quality pork that can be achieved by regulating the type and development of muscle fibers after birth.

Dietary supplementation with functional feed additives has been widely implemented to modulate muscle fiber properties, thereby improving pork quality. Many such feed additives are derived from phytochemicals or traditional food sources. Research evidence has indicated that dietary guanidinoacetic acid supplementation can enhance growth performance and skeletal muscle development in finishing pigs by modulating muscle fiber properties and regulating the expression of myogenic genes [4]. Thymol, a plant-derived monoterpene, can enhance meat quality by boosting oxidative metabolic capacity and promoting the muscle fiber types in finishing pigs [5]. Chlorophyllin, a semi-synthetic phytochemical, has been shown to facilitate the slow-twitch muscle fiber type by upregulating *PGC-1α* and subsequently enhancing *miR-208b* and *miR-499* expressions [6]. Resveratrol, a polyphenol found in grapes and berries, has been shown to improve quality parameters in finishing pigs by modulating muscle fiber properties and enhancing antioxidant capacity [7]. Furthermore, naringin, a flavonoid from citrus fruits, drives the conversion of skeletal muscle fibers from type II to type I via modulating the AMPK/PGC-1α pathway [8]. Therefore, muscle fibers can be effectively modulated through nutritional interventions using natural or nature-identical compounds.

L-malic acid (L-MA) is a key natural organic acid commonly distributed in various fruits and vegetables. Various biotechnological approaches, including bacterial fermentation and enzyme-mediated synthesis, can enhance its high-yield production [9]. Furthermore, L-MA serves as an essential intermediate in cellular metabolism, plays a crucial role in mitochondrial ATP production, and possesses antioxidant activity. Evidence from multiple studies suggests that dietary L-MA can enhance animal growth performance, strengthen antioxidant capacity, and improve meat quality [10,11]. Additionally, it has been found to promote a higher proportion of oxidative fiber types in muscle [12]. Earlier findings from our research highlighted that Taoyuan black pigs and Xiangcun black pigs have higher proportions of slow-twitch fibers and greater water-holding capacity than Duroc pigs, potentially due to higher muscle MA level [13]. Collectively, these observations imply a potential role for dietary L-MA in modulating the transition of myofiber types. Despite these findings, the effects of dietary L-MA on nursery piglets and the underlying mechanisms of myofiber type transformation remain unclear. Consequently, the present study was designed to evaluate the impacts of L-MA supplementation levels on the growth performance, carcass characteristics, and myofiber type composition in nursery piglets. Furthermore, the mechanisms were explored from the perspectives of muscle growth, antioxidant capacity, energy metabolism, and mitochondrial biogenesis. This study elucidated how dietary L-MA modulates myofiber type composition and provided evidence supporting its application as a functional nutrient to produce high-quality meat in swine production.

## 2. Results

### 2.1. Growth Performance

Table 1 summarizes the effects of L-MA on nursery piglets’ growth performance. Piglets fed with 0.5% and 2% L-MA exhibited higher final body weight (BW) and average daily gain (ADG) than those in the control (Con) group (*p* < 0.05). In addition, 0.5–2% L-MA increased average daily feed intake (ADFI) throughout the trial (*p* < 0.05). Furthermore, L-MA supplementation linearly enhanced final BW, ADG, and ADFI. Moreover, quadratic effects (*p* < 0.05) on ADFI were observed as dietary L-MA levels increased.

### 2.2. Carcass Traits

As presented in Table 2, piglets fed diets containing 0.5–2% L-MA had greater carcass weight (*p* < 0.05) than those in the Con group. The 0.5% L-MA group showed greater straight and oblique carcass length, whereas the 0.25% and 2% L-MA groups exhibited increased loin-eye muscle area relative to the Con group (*p* < 0.05). Moreover, increasing L-MA levels linearly enhanced (*p* < 0.05) carcass weight, oblique carcass length, as well as loin-eye muscle area of nursery piglets.

### 2.3. Muscle Characteristics

The impacts of L-MA on the muscle characteristics of nursery piglets are listed in Table 3. At 45 min post-mortem, the pH value in the *Longissimus thoracis* (LT) muscle was elevated (*p* < 0.05) in piglets receiving 0.25% L-MA relative to the Con group. In addition, at 24 h post-mortem, all L-MA-treated groups exhibited decreased pressure loss and drip loss (*p* < 0.05), and cooking loss was lower (*p* < 0.05) in the 1% L-MA group relative to the Con group. Moreover, L-MA (0.25–2%) tended to reduce shear force (*p* = 0.065) relative to the Con group. Furthermore, L-MA supplementation resulted in linear reductions in pressure loss, drip loss, and cooking loss (*p* < 0.05). Moreover, L-MA supplementation quadratically decreased (*p* < 0.05) pressure loss.

### 2.4. Antioxidant Function in Muscle

Figure 1 illustrates the impacts of L-MA on the muscle antioxidant function. Compared to the Con group, glutathione peroxidase (GSH-Px) activity in the LT muscle was increased in the 0.5–2% L-MA groups, superoxide dismutase (SOD) activity was elevated in the 0.25–2% L-MA groups, and malondial-dehyde (MDA) concentration was decreased in the 0.25% and 1% L-MA groups (*p* < 0.05; Figure 1A). In the *Psoas major* (PM) muscle (Figure 1B), 0.5% and 2% L-MA enhanced the GSH-Px activity, while 0.25% and 0.5% L-MA increased the total anti-oxidant capacity (T-AOC) activity relative to the Con group (*p* < 0.05). Additionally, supplementation with different doses of L-MA tended to increase SOD activity (*p* = 0.10) and decrease MDA concentration (*p* = 0.09).

### 2.5. Energy Metabolism Enzyme Activity in Plasma and Muscle

Figure 2 shows that plasma malate dehydrogenase (MDH) activity was elevated in the 0.5% and 1% L-MA groups, as well as plasma succinate dehydrogenase (SDH) activity in the 0.5% L-MA group, when compared with the Con group (*p* < 0.05; Figure 2A). In the LT muscle, SDH activity was elevated in the 0.5% and 2% L-MA groups relative to the 0.25% L-MA group, whereas 1% and 2% L-MA reduced lactate dehy-drogenase (LDH) activity (*p* < 0.05; Figure 2C). In addition, SDH activity in the PM muscle was elevated in the 0.5% and 2% L-MA groups (*p* < 0.05; Figure 2B).

### 2.6. Alteration of Muscle Fiber Type Composition in Skeletal Muscle

The influence of L-MA on the alteration of skeletal muscle’s fiber type composition is depicted in Figure 3. Relative to the Con group, dietary 0.5% and 1% L-MA supplementation resulted in a higher proportion of oxidative fibers while concurrently resulting in a lower proportion of glycolytic fibers in the LT muscle (*p* < 0.05; Figure 3A). Moreover, 0.5–2% L-MA enhanced the percentage of oxidative fibers, whereas it reduced the percentage of glycolytic fibers in the PM muscle (*p* < 0.05; Figure 3B).

Furthermore, relative to the Con group, 0.5–2% L-MA upregulated myosin heavy chain I (*MyHCI*) expression in the LT muscle, while 2% L-MA downregulated myosin heavy chain II (*MyHCIIx*) and myosin heavy chain IIb (*MyHCIIb*) expressions (*p* < 0.05; Figure 3C). Moreover, relative to the Con group, dietary 0.5%, 1%, and 2% L-MA upregulated (*p* < 0.05) *MyHCI* and myosin heavy chain IIa (*MyHCIIa*) expressions in the PM muscle (Figure 3D).

### 2.7. The Expression Profiles of Genes Involved in the Regulation of Skeletal Muscle Growth

Figure 4A illustrates the gene expression levels associated with skeletal muscle growth in the LT muscle. Compared to the Con group, 0.5% and 2% L-MA upregulated myogenic factor 6 (*Myf6*) and forkhead box protein O1 (*FOXO1*) expressions, while 0.5–2% L-MA downregulated SRY-box transcription factor 6 (*SOX6*) expression (*p* < 0.05). In addition, pigs fed 0.5% and 2% L-MA showed higher (*p* < 0.05) myogenic differentiation (*MyoD*) expression than those fed 0.25% L-MA.

Figure 4B illustrates the gene expression levels associated with skeletal muscle growth in the PM muscle. Relative to the Con group, pigs fed 0.5% and 2% L-MA showed upregulated *Myf6* expression (*p* < 0.05), while 0.5% L-MA showed upregulated *FOXO1* expression and downregulated *SOX6* expression (*p* < 0.05).

### 2.8. Expression Levels of Genes Associated with Antioxidant Capacity in Skeletal Muscle

Figure 5A depicts the expression levels of genes associated with antioxidant capacity in the LT muscle. Different doses of L-MA upregulated *SOD2* and *GSH-Px* expressions, while 0.5–2% L-MA upregulated *SOD1* and *GST* expressions in comparison with the Con group (*p* < 0.05). Additionally, 0.5% and 2% L-MA upregulated *CAT* expression relative to the Con group (*p* < 0.05). Moreover, *Keap1* expression was downregulated (*p* < 0.05) in nursery piglets receiving 0.25–1% L-MA compared to those in the Con group.

Figure 5B displays the gene expression profiles associated with antioxidant capacity in the PM muscle. All levels of dietary L-MA upregulated (*p* < 0.05) *SOD1* expression, and 0.5% L-MA upregulated (*p* < 0.05) *GSH-Px* and *GST* expressions relative to the Con group. Additionally, relative to the Con group, *SOD2* expression was upregulated (*p* < 0.05) in the 0.25% and 0.5% L-MA groups, as well as *CAT* expression in the 0.25%, 0.5%, and 2% L-MA groups, and *NRF2* expression in the 1% and 2% L-MA groups.

### 2.9. Mitochondrial Abundance in Skeletal Muscle

The influence of L-MA on skeletal muscle’s mitochondrial abundance is displayed in Figure 6. Relative to the Con group, different doses of L-MA resulted in a higher relative intensity of TOM20 in the LT muscle (*p* < 0.05; Figure 6A). Moreover, 0.5–2% L-MA enhanced the relative intensity of TOM20 in the PM muscle (*p* < 0.05; Figure 6B).

### 2.10. Gene Expression Levels Related to Mitochondrial Biogenesis in Skeletal Muscle

Figure 7A illustrates the expression levels of genes involved in mitochondrial biogenesis in the LT muscle. Relative to the Con group, the expression of cytochrome c oxidase (*COX*) *4* in the 0.5–2% L-MA groups was upregulated (*p* < 0.05), as well as cytochrome c (*Cytc*) in the 0.25% and 1% L-MA groups, polymerase gamma (*POLG*) in the 0.5% and 1% L-MA groups, and *COX1* in the 1% and 2% L-MA groups. Furthermore, analysis of genes involved in the mitochondrial biogenesis pathway revealed that AMP-activated protein kinase (*AMPK*) *α1* expression in the 0.25% and 2% L-MA groups was upregulated (*p* < 0.05), as well as nuclear respiratory factor 1 (*NRF1*) expression in the 0.5% and 2% L-MA groups, and sirtuin 1 (*Sirt1*) expression in the 0.25% L-MA group, relative to the Con group.

Figure 7B depicts the mitochondrial-biogenesis-related gene expression profiles in the PM muscle. Relative to the Con group, dietary supplementation with different doses of L-MA upregulated *COX4* and *POLG* expressions, 0.5–2% L-MA upregulated *Cytc* expression, and 0.5% and 2% L-MA upregulated transcription factor A mitochondrial (*TFAM*) expression (*p* < 0.05). Furthermore, gene expression related to the mitochondrial biogenesis pathway indicated that all L-MA treatments upregulated *NRF1* expression, 0.25–1% L-MA upregulated *AMPKα1* expression, 0.5% and 2% L-MA upregulated peroxisome-proliferator-activated receptor gamma coactivator 1-alpha (*PGC-1α*) expression, and 0.5% L-MA upregulated *Sirt1* expression, relative to the Con group (*p* < 0.05).

## 3. Discussion

The L-MA, as a green and efficient feed additive, has been utilized in livestock production to enhance growth performance, improve meat quality, and support health and immune function. However, limited research has evaluated the effects of dietary L-MA in nursery piglets. This study revealed that dietary L-MA supplementation effectively improved the growth of nursery piglets. Moreover, L-MA promoted a shift toward oxidative muscle fiber characteristics by enhancing antioxidant capacity, improving energy metabolism, and promoting muscle growth, thereby advancing the production of high-quality pork. Therefore, L-MA acts as a functional nutrient that enhances both livestock productivity and the nutritional properties of meat. This dual benefit aligns with the goals of improving food security, as more efficient production of high-quality protein helps meet global demand.

In this study, dietary L-MA supplementation exhibited higher ADG and ADFI, as well as increased final BW. Specifically, 0.5% and 2% L-MA enhanced final BW and ADG throughout the trial, indicating a positive impact of L-MA on BW gain during the nursery phase of pigs. Moreover, 0.5–2% L-MA could enhance ADFI, suggesting improved feed efficiency during the nursery period. These enhancements in growth performance corroborate the results reported by Qiu et al. [14], demonstrating that dietary supplementation with bio-fermented MA improved broiler productivity through increasing ADFI, ADG, and BW. Nevertheless, Zhang et al. [12] documented contrary evidence in weaned piglets and Yan et al. [11] in finishing pigs, where dietary L-MA supplementation did not result in significant alterations in growth performance. This discrepancy may stem from variations in the physiological stage at which L-MA was supplemented and the purity level of the L-MA preparation. Our study utilized L-MA with 99% purity, whereas the aforementioned studies employed L-MA products containing only 20% active compound.

In addition, the presence of both linear and quadratic effects further supports a dose-dependent response to dietary L-MA supplementation. Specifically, dietary L-MA linearly increased final BW, ADG, and ADFI throughout the experimental period, while also quadratically increasing ADFI. Collectively, these observations suggest that L-MA exerts a dose-responsive positive effect on the growth performance of nursery piglets.

Carcass traits are important indicators in pork production [15]. Carcass weight is associated with meat production and overall carcass quality. In our study, 0.5–2% L-MA increased carcass weight, suggesting that L-MA promotes overall body mass accretion. This observation aligns with the increased final BW observed in our study. Carcass length and loin-eye area are the key indicators for evaluating meat production efficiency and carcass value in livestock [16]. Our results showed that 0.5% L-MA supplementation increased both straight and oblique lengths of the carcass, while 0.25% and 2% L-MA increased the loin-eye area. These findings indicate that dietary L-MA could enhance skeletal growth and development. Similarly, Yan et al. [11] demonstrated that the inclusion of 0.5% and 2% L-MA in diets markedly enhanced carcass length and loin-eye area in finishing pigs. Additionally, regression analyses indicated that L-MA increased carcass weight and loin-eye area in a dose-dependent manner, demonstrating that dietary L-MA enhances skeletal growth and development with a dose–effect relationship.

Key consumer-oriented quality traits of pork encompass drip loss, color, pH, tenderness, and sensory characteristics [17]. Evaluations of pressure loss and drip loss provide reliable assessments of the muscle’s ability to retain water, a significant factor in meat quality [18]. Cooking yield is another indicator of water-holding capacity during cooking. A decrease in water-holding capacity leads to pronounced moisture exudation from pork, which reduces muscle weight and ultimately incurs economic losses [19]. Our results showed that L-MA effectively decreased both pressure loss and drip loss, while 1% L-MA also significantly decreased cooking loss. The observed reductions in drip and cooking losses imply that L-MA supplementation may enhance both water-holding capacity and cooking yield, thereby improving meat quality. Consistent with our findings, a previous study observed a decrease in drip loss in finishing pigs following L-MA supplementation [12]. In addition, the increased pH_45min_ suggests a slower rate of glycolysis and less lactic acid accumulation after slaughter, thereby enhancing water-holding capacity and reducing the occurrence of pale soft exudative meat. Moreover, the linear and quadratic reductions in drip loss, as well as the quadratic reduction in pressure losses, further indicate a dose-responsive improvement in water retention. Additionally, a trend toward decreased shear force suggests that L-MA may contribute to improved tenderness, as shear force correlates with meat tenderness [20].

The antioxidant status of muscle is closely linked to both meat color and nutritional value [21]. Studies have demonstrated that dietary inclusion of lycopene [22], *Inonotus obliquus* fermentation products [23], and grape seed proanthocyanidin extract [21] can improve the oxidative stability of meat and its color while reducing lipid oxidation. In this study, GSH-Px activity in the LT muscle of the 0.5–2% L-MA groups was elevated, as well as in the PM muscle of the 0.5% and 2% L-MA groups. Moreover, different doses of L-MA enhanced SOD activity in the LT muscle, whereas 0.25% and 0.5% L-MA increased T-AOC activity in the PM muscle. Additionally, MDA content in the LT muscle was reduced in the 0.25% and 1% L-MA groups, while a decreasing trend in MDA content was observed in the PM muscle following L-MA supplementation. Collectively, these observations imply that dietary L-MA can markedly improve the antioxidant capacity of skeletal muscle in nursery piglets, with potential implications for both animal health and pork quality. This may be partially related to the metabolic role of L-MA as a TCA cycle intermediate and to the transcriptional regulation of mitochondrial-biogenesis-related genes observed in this study. However, the exact mechanism remains to be elucidated.

The enhanced antioxidant capacity observed in muscle tissues in this study was linked to upregulation of antioxidant-related gene expression. For instance, different doses of dietary L-MA upregulated the expressions of *SOD2* and *GSH-Px*, while 0.5–2% L-MA upregulated *SOD1* and *GST* in the LT muscle. Furthermore, *CAT* expression in the LT muscle was upregulated by 0.5% and 2% L-MA supplementation. Notably, the expression of *Keap1*, which negatively regulates the NRF2-mediated antioxidant pathway, was downregulated in nursery piglets fed with 0.25–1% L-MA, suggesting activation of the NRF2-mediated antioxidant response. Similarly, in the PM muscle, L-MA supplementation (0.25–2%) upregulated *SOD1* expression, 0.5% L-MA upregulated *GSH-Px* and *GST* expressions, while 0.25% and 0.5% L-MA upregulated *SOD2* expression. Moreover, *CAT* expression was upregulated in the 0.25%, 0.5%, and 2% L-MA groups, whereas higher L-MA doses (1% and 2%) upregulated *NRF2* expression. These findings highlight that dietary L-MA improves antioxidant defense by modulating the NRF2 pathway. This enhances the muscle’s capacity to mitigate oxidative stress, which is critical for muscle integrity and meat quality. Moreover, these observations are consistent with earlier reports in finishing pigs, in which L-MA supplementation improved the antioxidant capacity of skeletal muscle [11].

Muscle fiber types differ in their metabolic functions, as slow fibers exhibit higher SDH and MDH activities, as well as lower LDH activity, compared to fast fibers [24,25]. The present study findings indicate that different doses of L-MA modulated key enzyme activities related to energy metabolism in both plasma and skeletal muscle. However, the biological interpretation of these changes differs between plasma and tissue. In the skeletal muscle, 0.5% and 2% L-MA supplementation increased the SDH activity in the LT muscle compared to the 0.25% L-MA group. In addition, 0.5% and 2% L-MA supplementation increased the SDH activity in the PM muscle compared with the 0.25% L-MA group. The SDH is widely recognized as a biomarker of oxidative capacity and mitochondrial density. Its upregulation indicates enhanced mitochondrial oxidative phosphorylation and a metabolic change toward aerobic energy production in muscle tissue. Conversely, 1% and 2% L-MA supplementation decreased LDH activity in the LT muscle. This suppression of LDH activity further supports a metabolic transition away from anaerobic glycolysis toward oxidative metabolism, consistent with the observed increase in SDH activity. 

In plasma, MDH activity was increased in nursery piglets fed 0.5% and 1% L-MA, and plasma SDH activity was increased in pigs fed 0.5% L-MA. Unlike muscle enzyme activities, circulating enzyme activities may reflect enzyme leakage from tissues or increased tissue turnover rather than a direct enhancement of muscle oxidative capacity. Therefore, these plasma data should be interpreted with caution and cannot be taken as direct evidence of improved muscle oxidative metabolism. Future studies measuring markers of muscle damage and turnover (e.g., creatine kinase and LDH isoenzymes) are needed to clarify the biological significance of these plasma enzyme changes. These findings highlight the potential of dietary L-MA supplementation to promote a favorable metabolic profile in pigs, supporting both muscle development and pork quality. These observations are similar to those of Zhang et al. [12], who found that L-MA can enhance energy metabolism in weaned piglets. However, Yan et al. [11] reported no significant effects in finishing pigs. These inconsistencies may be explained by differences in both the timing of L-MA supplementation and its chemical form. In addition, the specific underlying mechanisms require further investigation.

Myosin heavy-chain (MyHC) isoforms are the primary determinants of muscle fiber type [26], and the resulting fiber type composition largely determines meat quality traits such as color, texture, and flavor [27]. Relative to glycolytic fiber types, the predominant oxidized fiber types are higher in mitochondria and can facilitate oxidative metabolism, which may contribute to changes in meat color [27]. Our findings demonstrate that dietary L-MA induces a remodeling of muscle fiber profiles in nursery piglets, driving a transition from glycolytic toward oxidative fiber types in both the LT and PM muscles. Specifically, the immunofluorescence staining indicated that 0.5% and 1% L-MA supplementation increased the proportion of oxidative fibers while decreasing glycolytic fiber percentages in the LT muscle. Similarly, different doses of L-MA promoted oxidative fiber formation and reduced glycolytic fibers in the PM muscle. Moreover, gene expressions associated with muscle fiber types also indicated that L-MA modulates muscle fiber type composition. Within the LT muscle, 0.5–2% L-MA upregulated the *MyHCI* expression, whereas 2% L-MA downregulated *MyHCIIx* and *MyHCIIb* expressions. Meanwhile, L-MA supplementation upregulated *MyHCI* and *MyHCIIa* expressions in the PM muscle. In agreement with our findings, previous research has also reported that dietary L-MA may facilitate the shift from glycolytic to oxidative fibers in weaned piglets [12], as well as in finishing pigs [11].

The *MyoD*, *MyoG*, *Myf5*, and *Myf6* constitute regulatory factors that orchestrate muscle fiber development, overseeing their formation, differentiation, and functional optimization [28]. These regulatory genes modulate the isoform composition of *MyHC* by influencing gene expression [29]. *FOXO1* is a transcription factor involved in muscle metabolism and regulation of atrophy, and it plays a crucial role in driving the conversion toward oxidative muscle fibers [30], whereas Sox6 serves as a transcriptional inhibitor of oxidative muscle genes, and its regulation is critical for maintaining the glycolytic muscle phenotype [31]. In the current experiment, 0.5% and 2% L-MA upregulated *Myf6* and *FOXO1* expressions, while 0.5–2% L-MA downregulated *SOX6* expression in the LT muscle. Furthermore, 0.5% and 2% L-MA upregulated *Myf6* expression, while 0.5% L-MA upregulated *FOXO1* expression; however, 0.5% L-MA downregulated *SOX6* expression in the PM muscle. The changes in these gene expressions indicate that dietary L-MA supports skeletal muscle growth and fiber-type remodeling through coordinating the regulation of myogenic factors and transcriptional repressors.

Mitochondrial biogenesis is related to the transformation of muscle fiber types [32]. Moreover, mitochondria are also pivotal in influencing muscle fiber size and quantity [33]. TOM20 is a core receptor of the outer-mitochondrial translocase complex, and its protein abundance is highly correlated with mitochondrial biogenesis [34]. Our present study showed that different doses of L-MA elevated TOM20 intensity in the LT muscle, while 0.5–2% L-MA also enhanced TOM20 intensity in the PM muscle. These results demonstrate that dietary L-MA effectively boosts mitochondrial abundance.

Furthermore, mitochondrial biogenesis is regulated by multiple factors, including *AMPK*, *PGC-1α*, and *SIRT1* [35]. The AMPK/PGC-1α signaling pathway is a principal canonical pathway that regulates mitochondrial biogenesis, which is vital for maintaining mitochondrial function and facilitating the transformation of muscle fiber types [36]. Previous research has demonstrated that modulation of the AMPK/PGC-1α signaling pathway facilitates the conversion of skeletal muscle fibers from glycolytic (type II) to oxidative (type I) phenotypes [8,37]. The present findings show that 0.5–2% L-MA elevated the expression of mitochondrial marker genes (*COX4*) in the LT muscle, indicating enhanced mitochondrial content and function. Additionally, the *Cytc* expression was upregulated by 0.25% and 1% L-MA supplementation, while *POLG* was upregulated by 0.5% and 1% L-MA supplementation, both of which are essential for mitochondrial replication and electron transport chain activity. Moreover, higher L-MA levels (1% and 2%) upregulated *COX1* expression, a key component of complex IV in the respiratory chain. Notably, genes involved in the mitochondrial biogenesis regulatory pathway were also positively modulated. Moreover, 0.25% and 2% L-MA upregulated *AMPKα1* expression, while 0.5% and 2% L-MA upregulated *NRF1* expression. The *Sirt1*, a key deacetylase regulating mitochondrial biogenesis and metabolism, was significantly upregulated in the 0.25% L-MA group. Through transcriptional regulation of the AMPK/SIRT1/PGC-1α/NRF1 signaling pathway, these genes may have enhanced mitochondrial biogenesis and function, suggesting that dietary L-MA may promote mitochondrial formation and function via this canonical pathway.

Similar regulatory effects were observed in the PM muscle, where different doses of L-MA upregulated *COX4* and *POLG* expressions. The *Cytc* expression was upregulated by 0.5–2% L-MA supplementation. In addition, *TFAM*, a mitochondrial transcription factor critical for mtDNA replication, was upregulated by 0.5% and 2% L-MA supplementation. Furthermore, different doses of dietary L-MA consistently upregulated *NRF1* expression, while 0.25–1% L-MA upregulated *AMPKα1* expression. Notably, *PGC-1α*, the principal regulator of mitochondrial biogenesis [35], was significantly upregulated by 0.5% and 2% L-MA, while *Sirt1* expression was upregulated by 0.5% L-MA supplementation. Collectively, these findings indicate that dietary L-MA may be associated with the transcriptional regulation of mitochondrial-biogenesis-related genes and increased mitochondrial abundance in porcine skeletal muscle, possibly via the AMPK/SIRT1/PGC-1α/NRF1 signaling cascade. Mitochondrial biogenesis has been reported to improve oxidative capacity, energy metabolism, and muscle endurance. Consistent with this notion, the present study observed an increased proportion of oxidative fiber type and improvements in several meat quality traits, which may be associated with the increased mitochondrial abundance and upregulated expression of mitochondrial-biogenesis-related genes. However, direct evidence for enhanced mitochondrial biogenesis and its causal contribution to these phenotypic changes is still lacking. A limitation of this study is that the protein abundances of key enzymes in the AMPK/SIRT1/PGC-1α/NRF1 pathway were not determined. Future studies measuring these protein abundances are needed to elucidate the exact regulatory mechanism more precisely.

## 4. Materials and Methods

### 4.1. Experimental Design and Feeding Regimens

The study was designed with 40 castrated male Duroc × Landrace × Yorkshire nursery piglets (initial BW of 8.06 ± 0.43 kg) that were randomly allocated into one of five groups. Each group consisted of eight pigs (replicates), with one pig per replicate. Experimental animals were housed individually. The basal diet was supplemented with 0%, 0.25%, 0.5%, 1%, or 2% L-MA. The basal and other treatment diet formulations and nutrient levels were based on NRC (2012) recommendations (Table S1). After a 3-day acclimation period, the formal feeding experimental procedure was carried out for a period of 56 days. Water and feed were available *ad libitum* throughout the experimental period. The temperature of the experimental facility was maintained at 22–25 °C, and adequate ventilation was ensured throughout the trial. Concurrently, consistent access to lighting, temperature, humidity, and pen environmental conditions was ensured for all groups of pigs. The L-MA with a purity of 99% was obtained from Anhui Huaheng Biotechnology Co., Ltd. (Hefei, China).

### 4.2. Determination of Growth Performance

Daily feed intake was recorded for each pig. The BW of each pig was measured at both the start and end of the trial. The ADG, ADFI, and feed-to-gain ratio (F/G) were then calculated using Microsoft Excel 2021 (Microsoft Corp., Redmond, WA, USA). ADG was calculated as (final BW − initial BW)/experimental days, ADFI was calculated as total feed intake/experimental days, and F/G was calculated as daily feed intake/daily gain.

### 4.3. Sample Collection

Following 12 h of fasting, all pigs at 56 days of the trial were euthanized in accordance with standard procedures, which consisted of electrical stunning (120 V, 200 Hz) followed by exsanguination. Blood samples were drawn from the precaval vein into 10 mL tubes and centrifuged at 3500× *g* for 10 min at 4 °C to obtain plasma. Plasma samples were preserved at −20 °C for subsequent determination of energy metabolism enzymes. The carcasses (after removal of head, hooves, tail, and viscera) were harvested to assess carcass and muscle characteristics. The LT and PM muscle tissues were collected at 30 min post-mortem and preserved in 4% paraformaldehyde solution for immunofluorescence analysis. The LT and PM muscle samples were also collected for gene expression analysis and stored at −80 °C.

### 4.4. Measurement of Carcass Traits and Muscle Characteristics

Based on the NY/T 825-2004 protocol, carcass traits were assessed by dissecting the left side of each carcass. Hot carcass weight was measured to determine the dressing percentage as described previously [38]. Carcass straight length was taken as the straight-line distance between the anterior edge of the pubic symphysis and the anterior edge of the first cervical vertebra, while oblique length was taken as the diagonal distance from the anterior edge of the pubic symphysis to the junction of the first rib and the sternum. The backfat thickness was taken at the site between the sixth and seventh ribs with a Vernier caliper. The skin fat, bone, lean meat, and leaf fat were isolated and weighed to determine the percentages of skin fat, bone, and lean meat, as well as the leaf fat ratio, following the method described by Zhu et al. [39]. To determine the lion-eye muscle area, the LT muscle cross-sectional width and height were recorded with a Vernier caliper, and the area was calculated [40].

Measurements of muscle pH were conducted at 45 min and 24 h post-slaughter using a portable pH meter (Russell CD700; Russell pH Limited, München, Germany). The LT muscle’s redness (a*), yellowness (b*), and lightness (L*) were assessed at 45 min post-slaughter with a colorimeter (CR410; Konica Minolta Sensing, Tokyo, Japan). The LT muscle drip loss was determined following the method reported by Honikel [41] with slight modifications. Briefly, strip-shaped LT muscle samples (10 g) were weighed to obtain initial weight, suspended on metal hooks within sealed polyethylene bags, and stored for 24 h at 4 °C. Following the removal of surface moisture, samples were reweighed, and the drip loss was calculated using the previously described formula [41]. Furthermore, the procedures for determining cooking loss and shear force were conducted as described in previous studies [38,42]. Briefly, the LT muscle samples were sequentially immersed in a 75 °C water bath until their internal temperature reached 70 °C. The samples were then removed, cooled to room temperature, surface-dried with paper towels, and reweighed to determine the cooking loss percentage: [(fresh weight − cooked weight)/fresh weight] × 100. Moreover, cooked samples were cut into rectangular blocks along the muscle fiber orientation using a scalpel. Shear force was assayed with six replicates per sample using a texture analyzer (FTCTMS/PRO; FTC Corporation, Sterling, VA, USA) set at a preload force of 15 kg and a crosshead speed of 200 mm/min.

### 4.5. Determination of Antioxidant Function in Muscle

According to the manufacturer’s guidelines, the activities of antioxidant enzymes, including catalase (CAT, Catalog No. G0106W), GSH-PX (Catalog No. G0204W), SOD (Catalog No. G0102W), T-AOC (Catalog No. G0142W), and the concentration of MDA (Catalog No. G0110W) in LT and PM muscles were quantified using Grace kits (Grace Biotechnology Co., Ltd., Suzhou, China).

### 4.6. Plasma and Muscle Energy Metabolic Enzyme Activities Analysis

Based on the manufacturer’s protocols, the activities of energy metabolism enzymes, including MDH (Catalog No. G0821W), LDH (Catalog No. G0804W), and SDH (Catalog No. G0856W) in plasma, LT muscle, and PM muscle were evaluated using commercial kits (Grace Biotechnology Co., Ltd., Suzhou, China).

### 4.7. Myofiber Type Immunofluorescence Analysis

Immunofluorescence staining of LT and PM muscles was performed to evaluate myofiber type composition. Briefly, muscle tissues were embedded in paraffin (JB-P5, Wuhan Junjie Electronics Co., Ltd., Wuhan, China) and then sectioned into 10 μm using a pathology sectioning machine (RM2016, Leica Microsystems Shanghai Ltd., Shanghai, China). The sections were blocked with 5% phosphate buffer saline for 30 min, incubated with FAST/SLOW primary antibodies (diluted 1:1000, AiFang Biological Co., Ltd., Changsha, China) at 4 °C overnight, and subsequently treated with a ready-to-use secondary antibody (AiFang Biological Co., Ltd., Changsha, China). Finally, DAPI (diluted 1:500, C0060, Solarbio, Beijing, China) was applied for nuclear counterstaining, and the sections were incubated with a spontaneous fluorescence-quenching reagent (Solarbio, S2100; Beijing, China). Fluorescence images were captured with a Nikon Eclipse C1 microscope (Tokyo, Japan) and subsequently scanned using a 3D HISTECH Panoramic MIDI slide scanner (3D HISTECH, Budapest, Hungary). Slow-twitch (type I) and fast-twitch (type II) fiber percentages were determined with ImageJ software (version 1.54k, National Institutes of Health, Bethesda, MD, USA).

### 4.8. Immunohistochemistry of Translocase of Outer Mitochondrial Membrane 20 (TOM20) to Evaluate Mitochondrial Abundance

Immunohistochemical staining of TOM20 was conducted to evaluate mitochondrial content in muscle tissues. Briefly, paraffin-embedded samples of the LT and PM muscles were sectioned at 4 μm thickness, deparaffinized in xylene, and rehydrated with a series of graded ethanol. Antigen retrieval was carried out in 10 mM EDTA (pH 9.0). Afterwards, the sections were washed in Tris-Buffered Saline with Tween 20 (TBST) three times for 5 min and blocked in 10% donkey serum for 30 min at 37 °C. Next, the sections were incubated overnight at 4 °C with a primary antibody against TOM20 (diluted 1:100, 42406, CST, Danvers, MA, USA). After washing, the sections were incubated with an Alexa Fluor^®^488 donkey anti-rabbit IgG (H + L) secondary antibody (diluted 1:400, A21206, Thermo Fisher, Waltham, MA, USA) for 45 min at 37 °C. Nuclei were counterstained with DAPI (diluted 1:500, C0060, Solarbio, Beijing, China), and slides were mounted with fluorescent mounting medium (0100-01, Southern Biotech, Birmingham, AL, USA). Images were captured at 50× magnification with identical exposure settings using an Olympus BX53 microscope (Olympus, Tokyo, Japan) equipped with a 3D HISTECH Pannoramic SCAN II scanner (3D HISTECH, Budapest, Hungary). Quantitative analysis of mitochondrial abundance was performed using ImageJ software (National Institutes of Health, Bethesda, MD, USA).

### 4.9. Target Gene Expression Analysis

The total RNA was extracted from LT and PM muscle samples using the TRIGene Reagent (GenStar BioSolutions Co., Ltd., Beijing, China) following the company’s protocols. RNA concentration was quantified with a NanoDrop ND-2000 spectrophotometer (Thermo Fisher Scientific, Waltham, MA, USA). RNA integrity was assessed by agarose gel electrophoresis. Subsequently, 1000 ng of the extracted RNA was reverse-transcribed into cDNA with StarScript III All-in-one RT Mix with gDNA Eraser (GenStar BioSolutions Co., Ltd., Beijing, China) for quantitative PCR analysis. RT-PCR analysis was conducted with a LightCycler^®^ 480II Real-Time PCR System (Roche, Basel, Switzerland) and 2 × HS Taq Universal SYBR Green qPCR Master Mix (Richamp Biology, Changsha, China). Specific primers for the target genes (listed in Table S3) were designed by Sangon Biotech Co., Ltd. (Shanghai, China). Each 10 μL RT-qPCR reaction mixture contained 0.25 μL of each forward and reverse primer, 5.0 μL of SYBR^®^ Green Premix, 2.0 μL of cDNA template, and 2.5 μL of ddH_2_O. PCR amplification was conducted according to the SYBR Green Premix guidelines. *β-actin* acted as the internal control, and relative gene expression between groups was quantified using the 2^−ΔΔCt^ method [43].

### 4.10. Statistical Analysis

A total of 40 pigs (*n* = 8 per group) were used in this study. An individual pig was considered the experimental unit. All data were analyzed using R software (version 4.4.1, R Core Team, 2022) and were presented as means ± SEM. Data normality was verified using the Shapiro–Wilk test, and homogeneity of variance was assessed using Levene’s test prior to the one-way ANOVA. The statistical model is expressed as follows:

*Yij* = *μ* + *Ti* + *εij*,

where *Yij* represents the observed value for the *j*-th replicate within the *i*-th treatment group, *μ* is the overall mean, *Ti* is the fixed effect of the *i*-th treatment (*i* = 1 to 5), and *εij* is the random residual error. Growth performance and carcass characteristics were further evaluated using linear and quadratic regression analyses to assess dose-dependent trends among the five treatment groups. Statistically significant results were determined at *p* < 0.05, and tendencies were considered at 0.05 ≤ *p* < 0.10. Tukey’s post hoc test was performed to examine significant differences among groups. The obtained statistical results were plotted using GraphPad Prism 9.5 (GraphPad Software Inc., San Diego, CA, USA).

## 5. Conclusions

To summarize, our findings highlight that dietary L-MA supplementation enhances ADG, ADFI, final BW, and muscle characteristics (reduced pressure loss, drip loss, and cooking loss) in nursery piglets by enhancing antioxidant capacity, stimulating oxidative metabolism, and promoting slow muscle fiber formation. These effects might be achieved by possibly modulating the AMPK/PGC-1α signaling cascade and mitochondrial biogenesis. These findings not only highlight the potential of L-MA as a functional feed additive but also offer new perspectives on the nutritional regulation of muscle development and meat quality of pigs. Importantly, L-MA represents a scalable, sustainable, and nature-identical functional nutrient by improving muscle characteristics that are known to be predictive of final pork quality. Our work not only advances the nutritional regulation of muscle development in growing piglets but also supports the application of L-MA as a functional additive in swine production systems, ultimately serving the goal of producing high-quality pork.

## Figures and Tables

**Figure 1:**
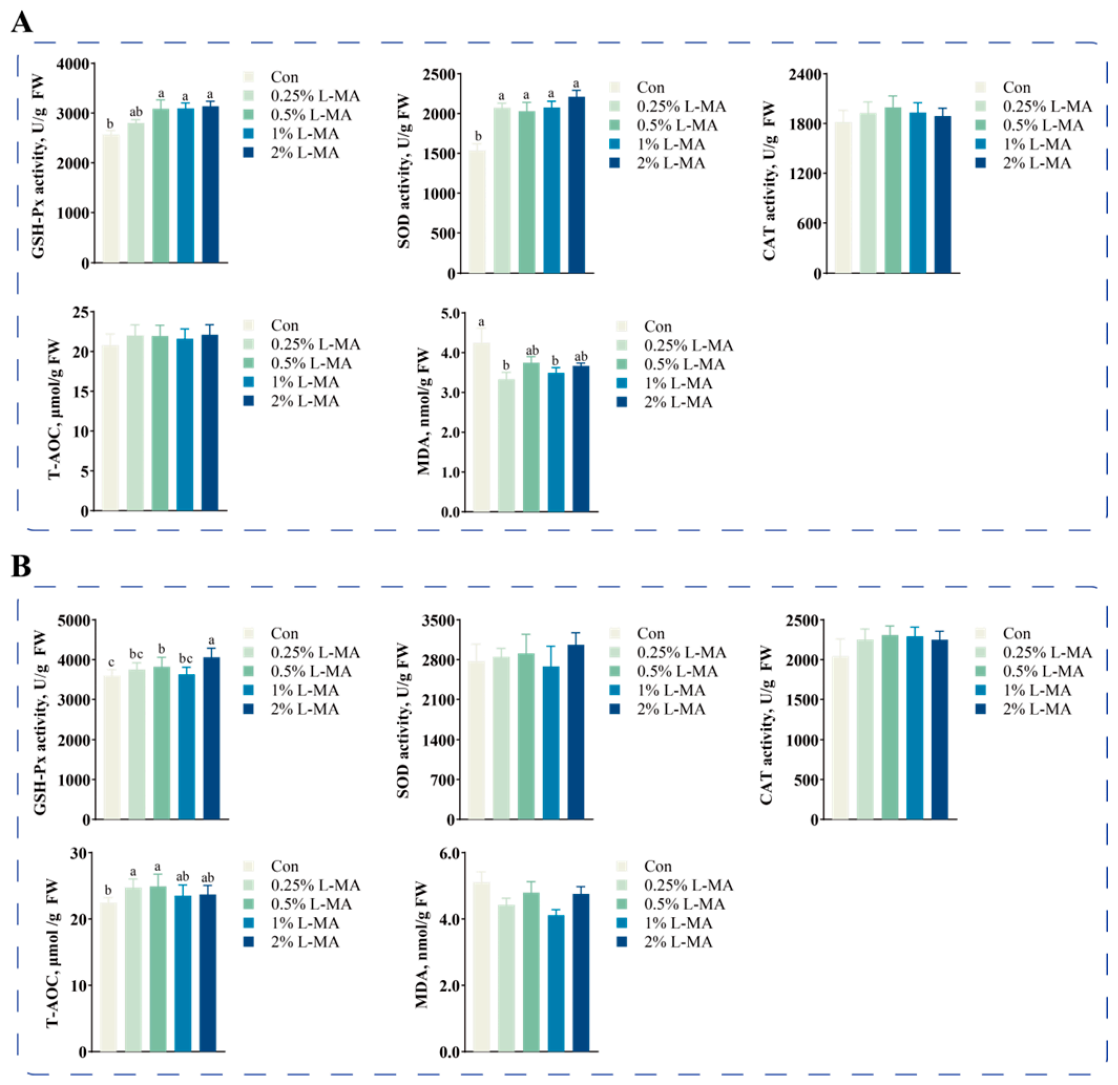
Effects of L-MA on the antioxidant function in the *Longissimus thoracis* muscle (**A**) and *Psoas major* muscles (**B**) of nursery piglets. Data are expressed as means ± SEM (*n* = 8). Different lowercase letters above bars indicate significant differences (*p* < 0.05). Enzyme activities in muscle tissues are expressed per gram of tissue (wet basis; U/g FW, nmol/g FW or μmol/g FW).

**Figure 2:**
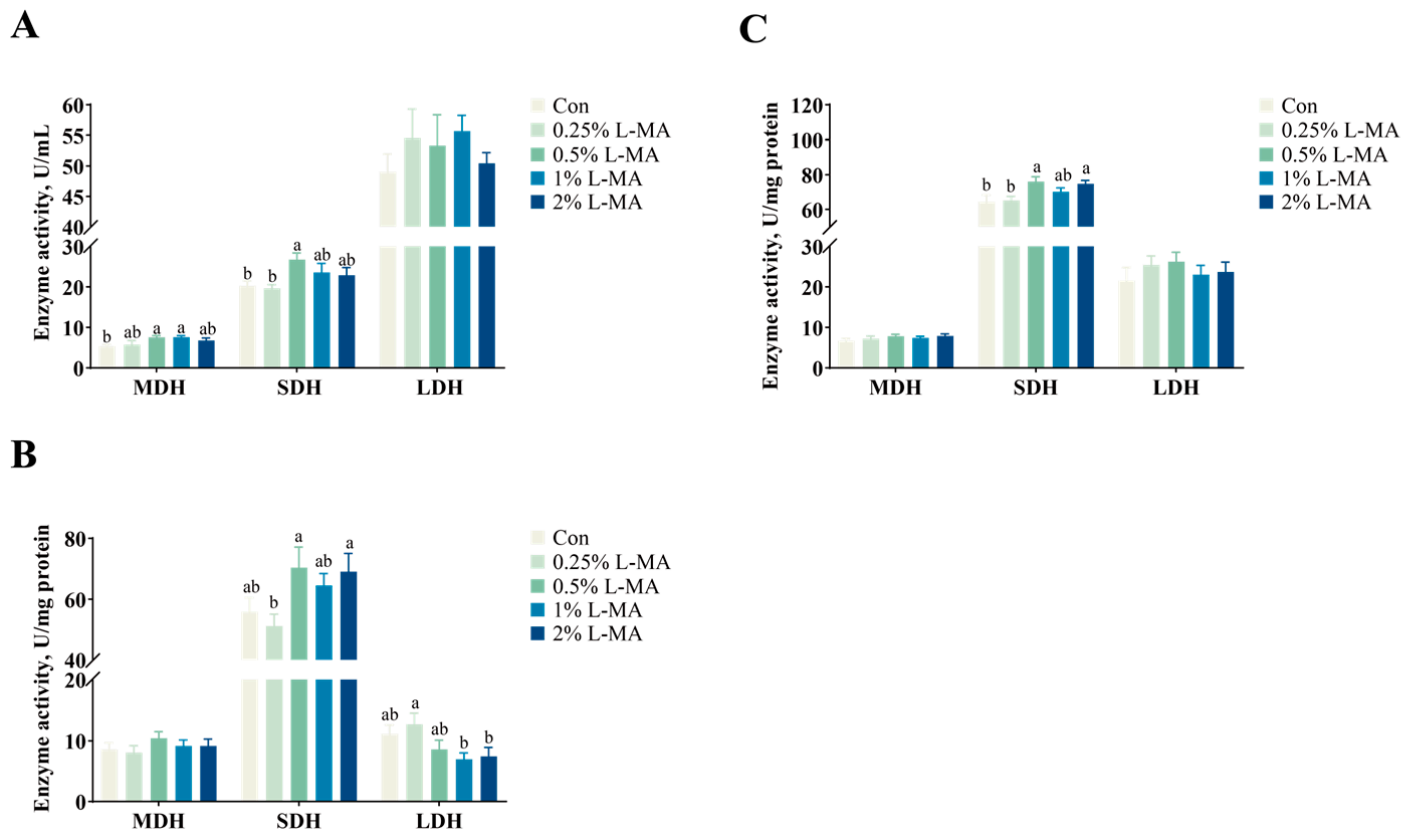
Effects of L-MA on energy metabolic enzyme activities in plasma (**A**), *Longissimus thoracis* muscle (**B**), and *Psoas major* muscle (**C**) of nursery piglets. Data are expressed as means ± SEM (*n* = 8). Different lowercase letters above bars indicate significant differences (*p* < 0.05). Enzyme activities in plasma are expressed as U/mL, and those in muscle tissues are expressed as U/mg protein.

**Figure 3:**
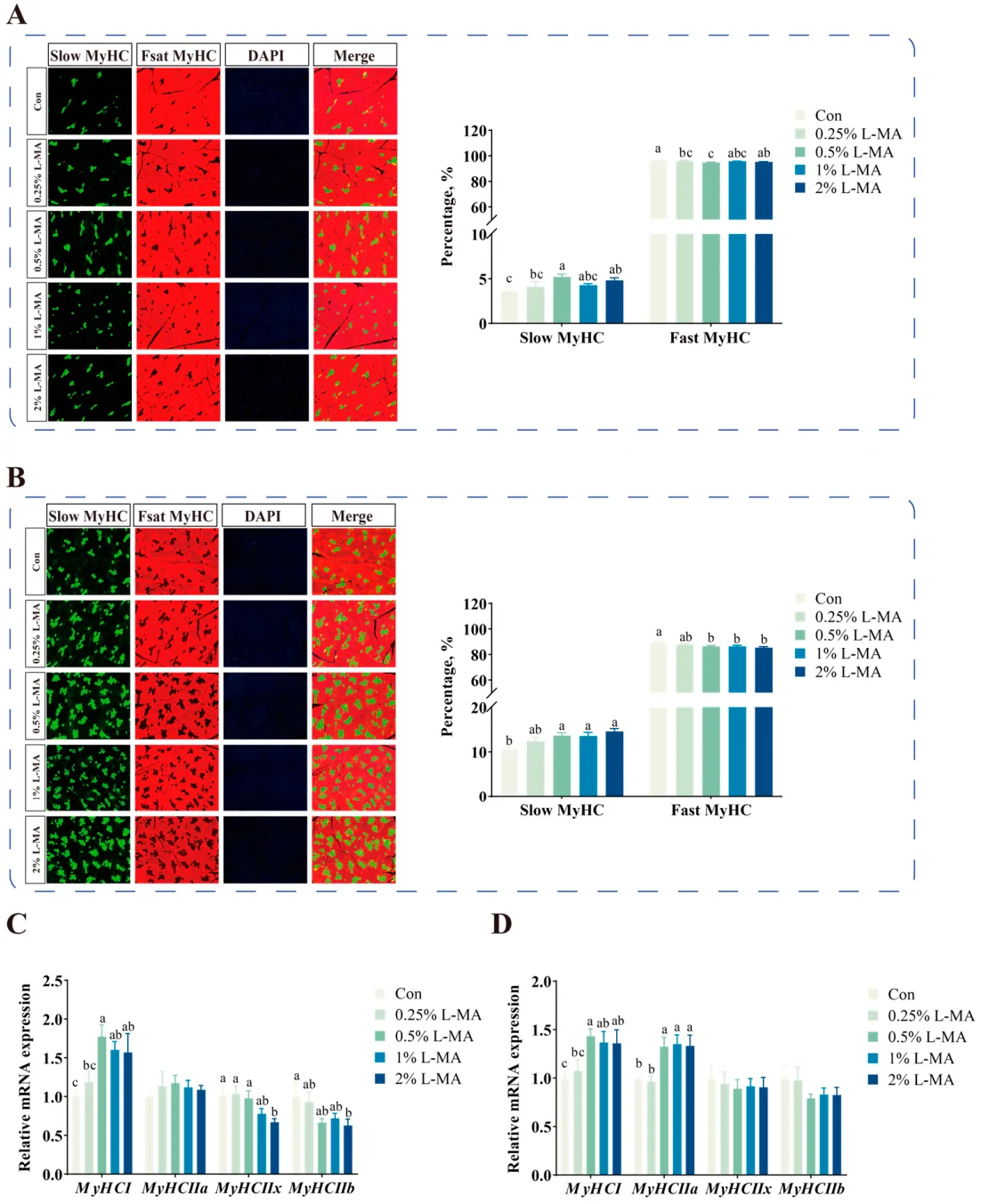
Effects of L-MA on muscle fiber type composition in the skeletal muscle of nursery piglets. Immunofluorescence analysis of the *Longissimus thoracis* (**A**) and *Psoas major* muscles (**B**) (green: oxidative fibers; red: glycolytic fibers; scale bar = 200 μm). The mRNA expression of myosin heavy chains (MyHCs) in the *Longissimus thoracis* (**C**) and *Psoas major* muscles (**D**). Data are expressed as means ± SEM (*n* = 8). Different lowercase letters above bars indicate significant differences (*p* < 0.05).

**Figure 4:**
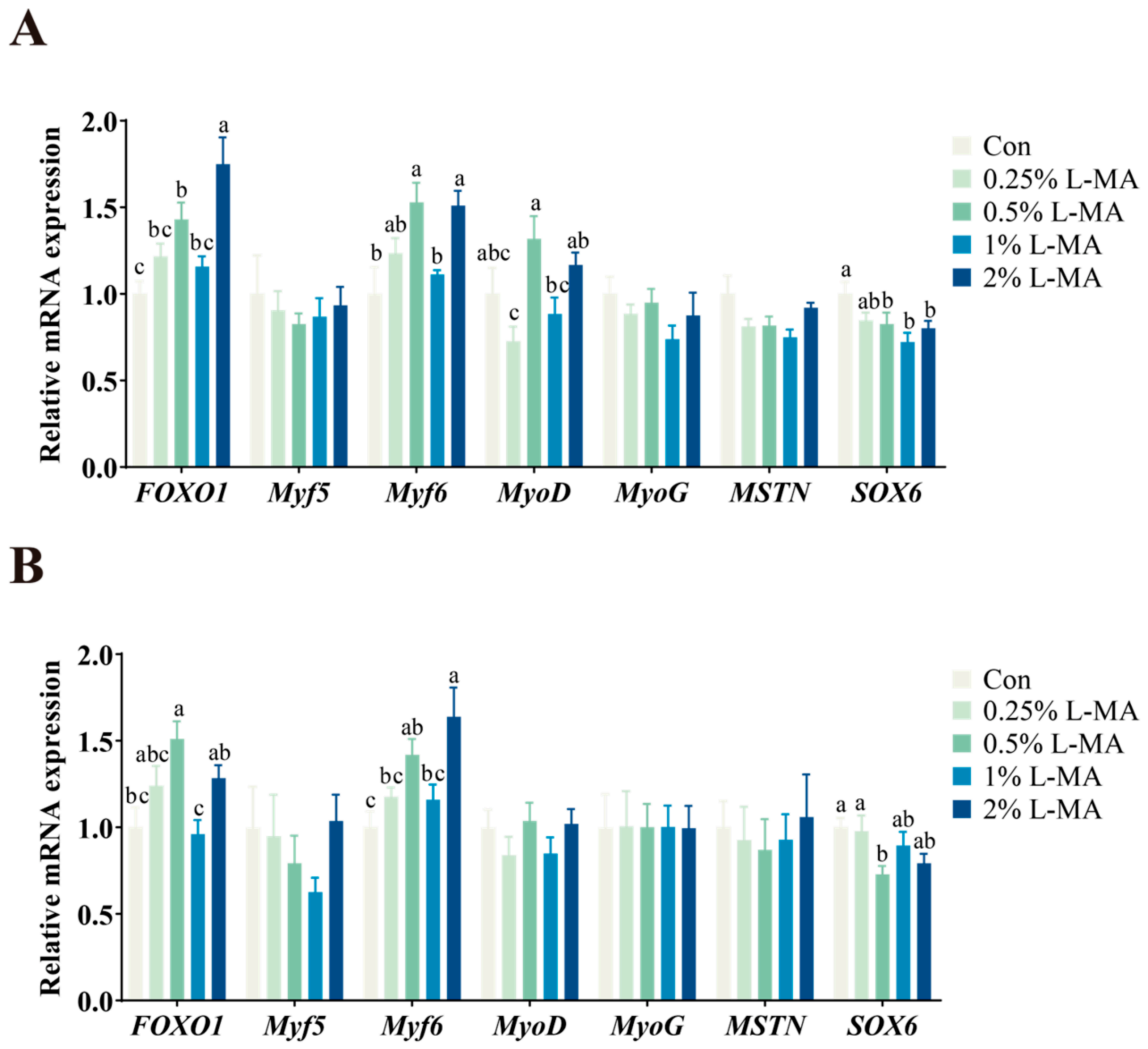
Effects of L-MA on the gene expression levels related to skeletal muscle growth and development in the *Longissimus thoracis* (**A**) and *Psoas major* muscles (**B**) of nursery piglets. Data are expressed as means ± SEM (*n* = 8). Different lowercase letters above bars indicate significant differences (*p* < 0.05).

**Figure 5:**
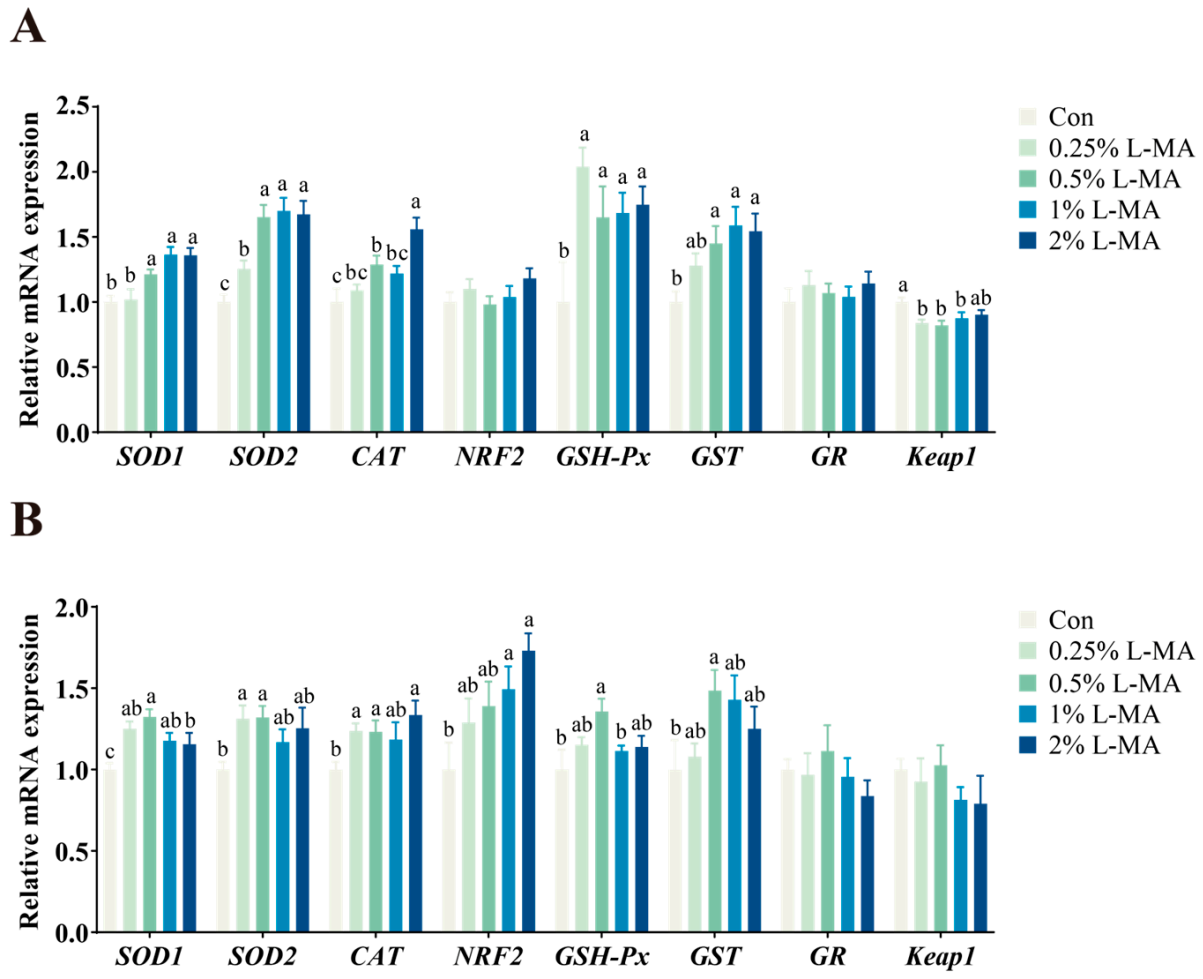
Effects of L-MA on the gene expression levels related to antioxidant capacity in the *Longissimus thoracis* (**A**) and *Psoas major* (**B**) muscles of nursery piglets. Data are expressed as means ± SEM (*n* = 8). Different lowercase letters above bars indicate significant differences (*p* < 0.05).

**Figure 6:**
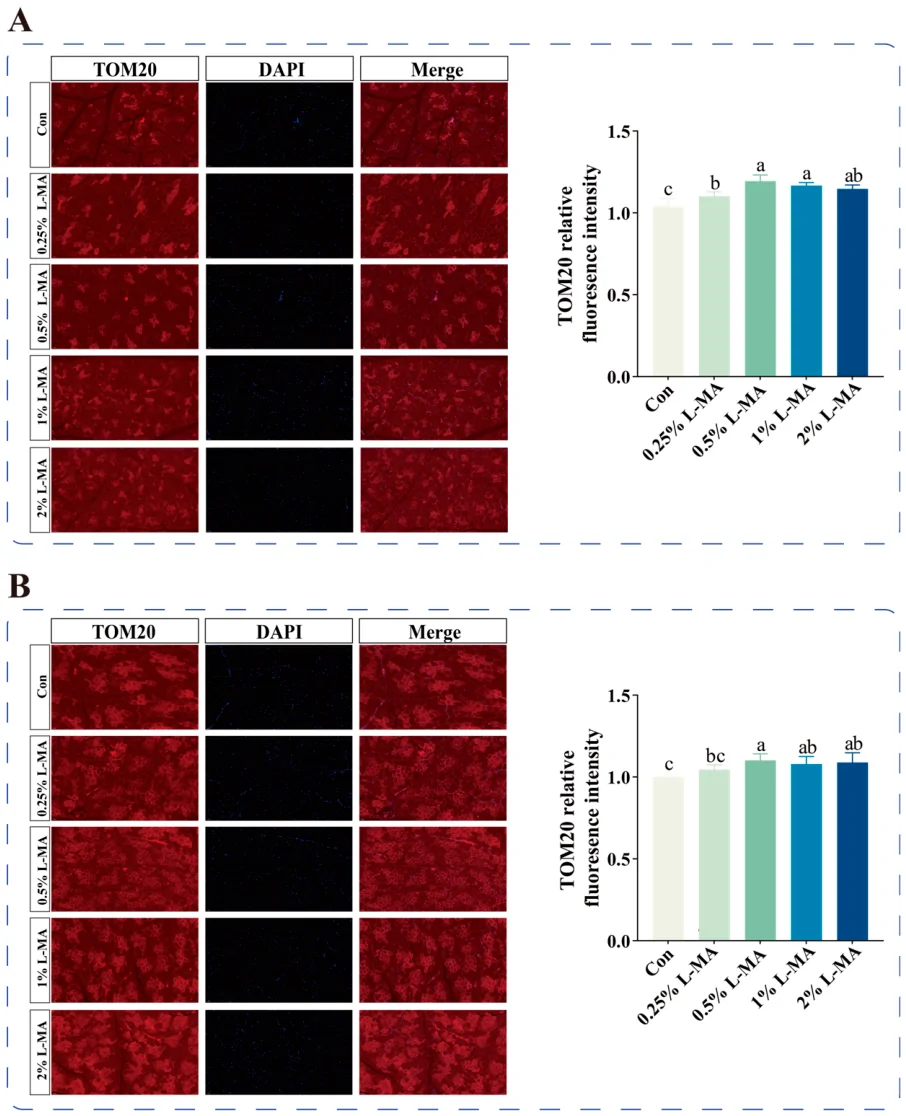
Effects of L-MA on mitochondrial content in the skeletal muscle of nursery piglets. Immunohistochemical detection of TOM20 in *Longissimus thoracis* (**A**) and *Psoas major* muscles (**B**). TOM20-positive mitochondria appear as red in the cytoplasm. Scale bar: 50 μm. Quantification of mitochondrial abundance was based on the percentage of TOM20-positive area. Data are expressed as means ± SEM (*n* = 8). Different lowercase letters above bars indicate significant differences (*p* < 0.05).

**Figure 7:**
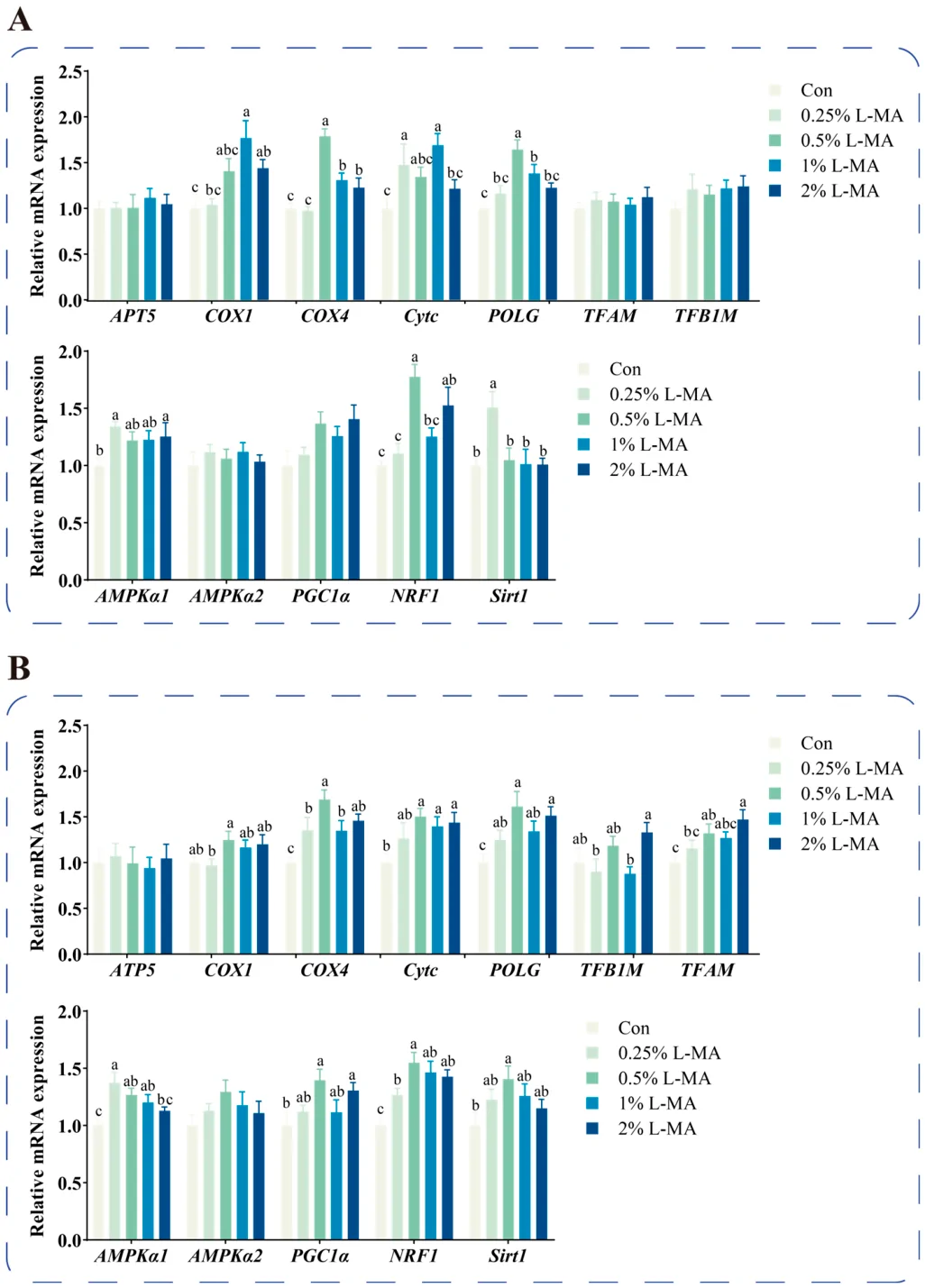
Effects of L-MA on the gene expression levels related to mitochondrial biogenesis in the *Longissimus thoracis* (**A**) and *Psoas major* (**B**) muscles of nursery piglets. Data are expressed as means ± SEM (*n* = 8). Different lowercase letters above bars indicate significant differences (*p* < 0.05).

**Table 1:** Effects of L-MA on the growth performance of nursery piglets.

Item	Con	L-MA, %				SEM	*p*-Values		
		0.25	0.5	1	2		ANOVA	Linear	Quadratic
Initial BW, kg	8.05	8.05	8.07	8.02	8.09	0.066	0.998	0.990	0.858
Final BW, kg	39.75 ^b^	40.03 ^b^	43.51 ^a^	41.76 ^ab^	43.31 ^a^	0.498	0.030	0.010	0.239
ADG, kg/d	0.56 ^c^	0.58 ^bc^	0.64 ^a^	0.61 ^abc^	0.62 ^ab^	0.009	0.029	0.018	0.118
ADFI, kg/d	1.13 ^c^	1.16 ^bc^	1.23 ^a^	1.21 ^ab^	1.20 ^ab^	0.011	0.006	0.004	0.043
feed-to-gain ratio	1.99	2.02	1.91	1.94	1.92	0.168	0.161	0.063	0.773

Note: Data are presented as means ± SEM (*n* = 8). Different superscript letters in the same row indicate significant differences (*p* < 0.05).

**Table 2:** Effects of L-MA on carcass traits of nursery piglets.

Item	Con	L-MA, %				SEM	*p*-Values		
		0.25	0.5	1	2		ANOVA	Linear	Quadratic
Dressing percentage, %	68.48	69.68	70.16	70.07	69.46	0.313	0.462	0.290	0.110
Carcass weight, kg	13.02 ^b^	13.36 ^b^	15.03 ^a^	14.36 ^a^	14.95 ^a^	0.187	<0.001	<0.001	0.154
Backfat thickness, mm	10.38	13.23	12.20	11.58	12.62	0.383	0.165	0.303	0.320
Straight carcass length, cm	75.14 ^b^	74.50 ^b^	79.92 ^a^	76.90 ^b^	77.00 ^b^	0.518	0.005	0.095	0.101
Oblique carcass length, cm	68.28 ^bc^	67.71 ^c^	71.10 ^a^	70.57 ^ab^	69.73 ^abc^	0.413	0.033	0.048	0.185
Skin and fat percentage, %	18.23	19.77	19.19	19.36	19.88	0.243	0.215	0.093	0.522
Bone percentage, %	18.79	18.51	18.80	19.31	19.04	0.181	0.713	0.315	0.873
Lean meat percentage, %	61.52	60.68	60.92	60.36	60.46	0.209	0.435	0.099	0.543
Leaf fat ratio, %	0.20	0.21	0.20	0.21	0.22	0.005	0.836	0.350	0.820
Loin-eye muscle area, cm^2^	11.84 ^c^	13.85 ^ab^	12.21 ^bc^	13.25 ^bc^	15.10 ^a^	0.320	0.005	0.007	0.342

Note: Data are presented as means ± SEM (*n* = 8). Different superscript letters in the same row indicate significant differences (*p* < 0.05).

**Table 3:** Effects of L-MA on *Longissimus thoracis* muscle characteristics of nursery piglets.

Item	Con	L-MA, %				SEM	*p*-Values		
		0.25	0.5	1	2		ANOVA	Linear	Quadratic
45 min after slaughter									
pH_45min_	6.37 ^b^	6.63 ^a^	6.45 ^b^	6.45 ^b^	6.46 ^b^	0.024	0.008	0.972	0.141
a*	20.77	21.15	20.51	20.92	20.52	0.148	0.641	0.501	0.684
b*	5.66	5.75	5.78	5.67	5.67	0.105	0.996	0.955	0.724
L*	43.94	43.52	42.95	43.48	43.72	0.180	0.523	0.705	0.114
24 h after slaughter									
pH_24h_	5.32	5.30	5.31	5.32	5.29	0.008	0.537	0.395	0.743
a*	18.75	19.04	18.99	19.19	19.61	0.170	0.599	0.115	0.722
b*	9.18	9.19	9.08	9.22	9.21	0.107	0.995	0.903	0.823
L*	53.09	52.64	52.95	52.88	53.10	0.323	0.993	0.911	0.728
Pressure loss, %	22.28 ^a^	18.81 ^b^	19.07 ^b^	17.70 ^b^	18.95 ^b^	0.497	0.038	0.025	0.046
Drip loss, %	3.41 ^a^	2.23 ^b^	2.13 ^b^	1.91 ^b^	1.89 ^b^	0.170	0.019	0.004	0.093
Cooking loss, %	33.54 ^a^	30.15 ^ab^	33.17 ^a^	29.58 ^b^	30.92 ^ab^	0.433	0.013	0.023	0.612
Shear force, N	53.49	44.15	48.00	46.44	45.86	1.097	0.065	0.095	0.178

Note: Data are presented as means ± SEM (*n* = 8). Different superscript letters in the same row indicate significant differences (*p* < 0.05).

## Data Availability

The original contributions presented in this study are included in the article/Supplementary Material. Further inquiries can be directed to the corresponding author(s).

## Supplementary Materials

The following supporting information can be downloaded at: https://www.mdpi.com/article/10.3390/molecules31193381/s1. Table S1: Composition and nutrient levels of the diet for the early nursery piglets (air-dried, %); Table S2: Composition and nutrient levels of the diet for the late nursery piglets (air-dried, %); Table S3: Primers sequences used for real time-PCR.

## Abbreviations

The following abbreviations are used in this manuscript:

AMPK	AMP-activated protein kinase
ATP5	ATP Synthase 5
CAT	catalase
COX	cytochrome c oxidase
Cytc	cytochrome c
FOXO1	forkhead box protein O1
GR	glutathione reductase
GSH-Px	glutathione peroxidase
GST	glutathione S-transferase
Keap1	kelch-like ECH-associated protein 1
LDH	lactate dehydrogenase
L-MA	L-malic acid
MDA	malondialdehyde
MDH	malate dehydrogenase
MyHCI	myosin heavy chain I
MyHCIIa	myosin heavy chain IIa
MyHCIIb	myosin heavy chain IIb
MyHCIIx	myosin heavy chain IIx
Myf5	myogenic factor 5
Myf6	myogenic factor 6
MyoD	myogenic differentiation
MyoG	myogenin
MSTN	myostatin
mtDNA	mitochondrial DNA
NRF1	nuclear respiratory factor 1
NRF2	nuclear factor erythroid 2-related factor 2
PGC1-α	peroxisome-proliferator-activated receptor gamma coactivator 1-alpha
POLG	polymerase gamma
SDH	succinate dehydrogenase
Sirt1	sirtuin 1
SOD	superoxide dismutase
SOX6	SRY-box transcription factor 6
T-AOC	total antioxidant capacity
TFAM	transcription factor A mitochondrial
TFB1M	transcription factor B1 mitochondrial
TOM20	Translocase of the outer mitochondrial membrane 20
Con	the pigs fed a basal diet
0.25% LMA, 0.5% L-MA, 1% L-MA, and 2% L-MA	the pigs fed a basal diet supplemented with 0.25% L-MA, 0.5% L-MA, 1% L-MA, and 2% L-MA, respectively
