# Supplementary material for: Dietary L-Malic Acid Enhances Growth Performance and Oxidative Myofiber Composition in Nursery Piglets
Source: Molecules. 2026 Sep 23;31(19):3381. doi: 10.3390/molecules31193381 (PMC13666365; doi:10.3390/molecules31193381)
Supplement: Supplementary file 1 [file molecules-31-03381-s001.zip › molecules-4521202-supplementary.pdf]

Table S1. Composition and nutrient levels of the diet for the early nursery piglets (air-dried, %).

Items	Control	L-malic acid			
		0.25%	0.5%	1%	2%
**Ingredients**					
Corn	64.40	63.90	63.40	62.50	60.50
Soybean meal	11.10	11.20	11.30	11.40	11.80
Extruded soybean	6.00	6.00	6.00	6.00	6.00
Fermented soybean meal	9.00	9.00	9.00	9.00	9.00
Wheat bran	3.00	3.00	3.00	3.00	3.00
Soybean oil	1.67	1.83	1.98	2.29	2.91
$CaHPO_4$	1.15	1.16	1.16	1.16	1.19
Limestone	0.75	0.73	0.74	0.74	0.69
Lysine	0.80	0.80	0.79	0.79	0.79
Methionine	0.30	0.30	0.30	0.30	0.30
Threonine	0.36	0.36	0.36	0.36	0.36
Tryptophan	0.09	0.09	0.09	0.08	0.08
Valine	0.13	0.13	0.13	0.13	0.13
NaCl	0.50	0.50	0.50	0.50	0.50
Choline chloride	0.15	0.15	0.15	0.15	0.15
L-malic acid	0.00	0.25	0.50	1.00	2.00
Premix[1]	0.60	0.60	0.60	0.60	0.60
Total	100.00	100.00	100.00	100.00	100.00
**Analyzed nutrient levels**					
Dry matter	87.06	87.15	87.41	87.61	87.50
Crude protein	19.26	19.61	19.66	19.94	19.74
Ether extract	3.61	3.80	4.42	5.15	5.27
Crude ash	4.73	4.59	4.52	4.63	4.36
Calcium	0.56	0.57	0.56	0.56	0.56
Total phosphorus	0.55	0.55	0.56	0.55	0.56
**Calculated nutrient levels[2]**					
Digestible energy, MJ/kg			14.23		
Metabolizable energy, MJ/kg			13.60		
SID[3] Lysine			1.40		
SID Methionine			0.50		
SID Methionine+ Cystine			0.80		
SID Threonine			0.90		
SID Tryptophan			0.30		

[1] Premix provides the following per kg of diets: vitamin A, 3 2500 IU; vitamin D_3, 10 000 IU; vitamin E, 80 IU; vitamin K_3, 10 mg; vitamin B_1, 10 mg; vitamin B_2, 25 mg; vitamin B_6, 8 mg; vitamin B_{12}, 0.075 mg; biotin, 0.6 mg; folic acid, 5 mg; nicotinamide, 100 mg; pantothenic acid, 50 mg; Cu (as $CuSO_4 \cdot 5H_2O$), 7.7 mg; Mn (as $MnSO_4 \cdot H_2O$), 7.3 mg; Zn (as $ZnSO_4 \cdot H_2O$), 109.3 mg; Fe (as $FeSO_4 \cdot H_2O$), 55.9 mg; I (as KI), 0.1 mg; Se (as Na_2SeO_3), 0.5 mg; Co (as $CoSO_4 \cdot H_2O$), 0.1 mg. [2] Calculated nutrient levels of the diets were obtained from the Feed Database in China (2020). [3] SID: standardized ileal digestibility.

Table S2. Composition and nutrient levels of the diet for the late nursery piglets (air-dried, %)

Items	Control	L-malic acid			
		0.25%	0.5%	1%	2%
**Ingredients**					
Corn	62.40	61.90	61.40	60.50	58.50
Soybean meal	26.00	26.10	26.20	26.30	26.70
Wheat bran	5.00	5.00	5.00	5.00	5.00
Soybean oil	2.67	2.83	2.98	3.28	3.87
$CaHPO_4$	1.05	1.06	1.07	1.08	1.09
Limestone	0.72	0.71	0.70	0.69	0.69
Lysine	0.56	0.55	0.55	0.55	0.55
Methionine	0.20	0.20	0.20	0.20	0.20
Threonine	0.25	0.25	0.25	0.25	0.25
Tryptophan	0.05	0.05	0.05	0.05	0.05
NaCl	0.50	0.50	0.50	0.50	0.50
Choline chloride	0.10	0.10	0.10	0.10	0.10
L-malic acid	0.00	0.25	0.50	1.00	2.00
Premix[1]	0.50	0.50	0.50	0.50	0.50
Total	100.00	100.00	100.00	100.00	100.00
**Analyzed nutrient levels**					
Dry matter	86.87	87.63	87.97	86.96	87.57
Crude protein	17.24	17.67	17.91	17.76	17.89
Ether extract	3.10	4.07	4.34	4.87	4.97
Crude ash	4.84	4.71	4.45	4.71	4.56
Calcium	0.52	0.52	0.52	0.51	0.51
Total phosphorus	0.54	0.55	0.55	0.55	0.56
**Calculated nutrient levels[2]**					
Digestible energy, MJ/kg			13.81		
Metabolizable energy, MJ/kg			13.39		
SID[3] Lysine			1.30		
SID Methionine			0.40		
SID Methionine+ Cystine			0.70		
SID Threonine			0.80		
SID Tryptophan			0.20		

[1] Premix provides the following per kg of diets: vitamin A, 3 2500 IU; vitamin D_3, 10 000 IU; vitamin E, 80 IU; vitamin K_3, 10 mg; vitamin B_1, 10 mg; vitamin B_2, 25 mg; vitamin B_6, 8 mg; vitamin B_{12}, 0.075 mg; biotin, 0.6 mg; folic acid, 5 mg; nicotinamide, 100 mg; pantothenic acid, 50 mg; Cu (as $CuSO_4 \cdot 5H_2O$), 6.2 mg; Mn (as $MnSO_4 \cdot H_2O$), 5.8 mg; Zn (as $ZnSO_4 \cdot H_2O$), 87.4 mg; Fe (as $FeSO_4 \cdot H_2O$), 42.8 mg; I (as KI), 0.08 mg; Se (as Na_2SeO_3), 0.4 mg; Co (as $CoSO_4 \cdot H_2O$), 0.08 mg. [2] Calculated nutrient levels of the diets were obtained from the Feed Database in China (2020). [3] SID: standardized ileal digestibility.

Table S3. Primers sequences used for real time-PCR.

Gene names	GenBank ID	Primer sequences (5′−3′)	Product size (bp)
β-actin	XM_021086047.1	F: GGCACCACACCTTCTACAACGAG<br>R: TCATCTTCTCACGGTTGGCTTTGG	102
AMPKα1	NM_001167633.1	F: CGGCAAAGTGAAGGTTGG<br>R: CCATCCGATACACCCACAT	123
AMPKα2	NM_214266.2	F: TGAGGTCGATATCTGGAGCTG<br>R: AGTGGCAACAGAACGATTGAG	151
ATP5G	XM_005653956.3	F: GTGAGTCAGTCACCTTGAGC<br>R: TCTGGCCTACTCAGGAAGGA	178
CAT	NM_214301.2	F: CAGATGAAGCATTGGAAGGAGC<br>R: TTGTCTCCTATCGGATTCCCAG	83
COX1	AJ950517.1	F: ATTATCCTGACGCATACACAGCA<br>R: GCAGATACTTCTCGTTTTGATGC	127
COX4	XM_021093706.1	F: CAGCGTCGTGAAGAGTGAGG<br>R: TTCGTGCTCCTGTTCATCTCG	219
Cytc	NM_001129970.1	F: TGCGGAGTGTTAAACTTTTCAGG<br>R: TGCCTTAACAGGCTAGTGAACA	191
FOXO1	NM_214014.3	F: TTCACCCAGCCCCAACTACC<br>R: GGAGGAGAGTCGGAAGTAAGTAAC	171
GR	XM_003483635.4	F: GTGAGCCGACTGAACACCAT<br>R: CAGGATGTGAGGAGCTGTGT	141
GSH-Px	NM_214201.1	F: GTGAATGGCGCAAATGCTCA<br>R: ATTGCGACACACTGGAGACC	126
GST	NM_214300.2	F: CCAACCCAGAAGACTGCTCA<br>R: CATTCAGGTGGGCTCTTCGT	102
Keap1	NM_001114671.1	F: AAACCGCCTCAACTCAGCAG<br>R: AGGTTCTGAATTTCTCTGCGG	148
MyHCI	NM_213855.2	F: CGTGGACTACAACATCATAGGC<br>R: CCTTCTCAACAGGTGTGTCG	146
MyHCIIa	NM_214136.1	F: CATTGAGGCCCAGAATAGGC<br>R: TGCTTCCGTCTTCACTGTCAC	127
MyHCIIx	NM_001104951.2	F: TTGACTGGGCTGCCATCAAT<br>R: GCCTCAATGCGCTCCTTTTC	111
MyHCIIb	NM_001123141.1	F: GACTCTGGCTTTCCTCTTTGC<br>R: GAGCTGACACGGTCTGGAAA	101
MyoD	NM_001002824.1	F: CTATGATGACCCGTGTTTCG<br>R: AGTGTTCCTCGGGCTTTAGG	101
MyoG	NM_001012406.1	F: AAACTACCTGCCCGTCCACCTC<br>R: GGTCCCCAGCCCCTTATCTTCC	112
Myf5	NM_001278775.1	F: GGATCAGCAACTCCGAGCAACC<br>R: GCACATGGTAGATGAGCCTGGAAC	126
Myf6	NM_001244672.1	F: GCTCGTGATGACTGCCAAGGAAG<br>R: CGATGGAAGAAAGGCACCGAAGG	80
MSTN	NM_214435.2	F: GCACCAAGCAAACCCCAGAGG<br>R: AGCACCCACAGCGATCTACTACC	143
mtDNA	XM_003483540.4	F: AACCGCTTTGATGGAGATGTAGG<br>R: GGTGAGGTCTGGCTGGTAGG	219
NRF-1	XM_021078998.1	F: CTGCTGCTGCTGCTGTGG<br>R:<br>AGGGTGAGATACAAAGGACAATAGC	174
NRF2	XM_021075131.1	F: GCCCCTGGAAGCGTTAAAC<br>R: GGACTGTATCCCCAGAAGGTTGT	67
PGC-1α	NM_213963.2	F: GCCCTCATTTGATGCACTG<br>R: AGCTGAGTGTTGGCTGGTG	150
POLG	XM_001927064.5	F: GACTCTGGCTGGCATGTTGG	152

		R: GCTGGCAGGCATCGTTGG	
Sirt1	NM_001145750.2	F: ACTCTCCCTCTTTTAGACCAAGC	149
		R: AAACCTGGACTCTCCATCGG	
SOX6	XM_021085349.1	F: GTGTTGTGTATCCTGGTGCTATTAC	147
		R: GGCTTCCGCTGTCTGTCTTC	
SOD1	NM_001190422.1	F: AGACCTGGGCAATGTGACTG	102
		R: GTGCGGCCAATGATGGAATG	
SOD2	NM_214127.2	F: TGTAACTGAGCGATACGCCG	99
		R: GGTATTCGGCGCTCCTACAA	
TFAM	NM_001130211.1	F: GCTCTCCGTTCAGTTTTGCG	187
		R: GGAAGTTCCCTCCACAGCTC	
TFB1M	NM_001128475.1	F: GCAAGCAGTGAAGCAGCTA	82
		R: CAGACTGCCAGCTTTCCTTAC	

Note: *AMPK*, AMP-activated protein kinase; *ATP5*, ATP synthase 5; *CAT*, catalase; *COX*, cytochrome c oxidase; *Cytc*, cytochrome c; *FOXO1*, forkhead box protein O1; *GR*, glutathione reductase; *GSH-Px*, glutathione peroxidase; *GST*, glutathione S-transferase; *Keap1*, kelch-like ECH-associated protein 1; *MyHCI*, myosin heavy-chain I; *MyHCIIa*, myosin heavy-chain IIa; *MyHCIIb*, myosin heavy-chain IIb; *MyHCIIx*, myosin heavy-chain IIx; *MyoD*, myogenic differentiation; *MyoG*, myogenin; *Myf5*, myogenic factor 5; *Myf6*, myogenic factor 6; *MSTN*, myostatin; m*tDNA*, mitochondrial DNA; *NRF1*, nuclear respiratory factor 1; *NRF2*, nuclear factor erythroid 2–related factor 2; *PGC-1α*, peroxisome proliferator-activated receptor gamma coactivator 1-alpha; *POLG*, polymerase gamma; *Sirt1*, sirtuin 1; *SOX6*, SRY-box transcription factor 60; *SOD*, superoxide dismutase; *TFAM*, transcription factor A mitochondrial; *TFB1M*, transcription factor B1 mitochondrial.
